# Gefitinib targets ZAP-70-expressing chronic lymphocytic leukemia cells and inhibits B-cell receptor signaling

**DOI:** 10.1038/cddis.2014.391

**Published:** 2014-10-02

**Authors:** R F Dielschneider, W Xiao, J-Y Yoon, E Noh, V Banerji, H Li, A J Marshall, J B Johnston, S B Gibson

**Affiliations:** 1 Department of Immunology, University of Manitoba, Winnipeg, MB, Canada; 2 Manitoba Institute of Cell Biology, Winnipeg, MB, Canada; 3 Department of Internal Medicine, University of Manitoba, Winnipeg, MB, Canada; 4 Department of Biochemistry and Medical Genetics, University of Manitoba, Winnipeg, MB, Canada

## Abstract

Chronic lymphocytic leukemia (CLL) can be divided into groups based on biomarkers of poor prognosis. The expression of the tyrosine kinase ZAP-70 (member of the Syk tyrosine kinase family) in CLL cells is associated with shorter overall survival in CLL patients. Currently, there is a lack of targeted therapies for patients with ZAP-70 expression in CLL cells. The tyrosine kinase inhibitor gefitinib has been shown to be effective at induce apoptosis in acute myeloid leukemia through inhibition of Syk. In this study, we sought to test the efficacy of gefitinib in primary human ZAP-70+ CLL cells. We demonstrate that gefitinib preferentially induces cell death in ZAP-70-expressing CLL cells with a median IC_50_ of 4.5 *μ*M. In addition, gefitinib decreases the viability of ZAP-70+ Jurkat T leukemia cells but fails to affect T cells from CLL patients. Western blot analysis shows gefitinib reduces both basal and B-cell receptor (BCR)-stimulated phosphorylation of Syk/ZAP-70, ERK, and Akt in ZAP-70+ CLL cells. Moreover, gefitinib inhibits the pro-survival response from BCR stimulation and decreases pro-survival proteins such as Mcl-1. Finally, ZAP-70 expression sensitizes Raji cells to gefitinib treatment. These results demonstrate that gefitinib specifically targets ZAP-70+ CLL cells and inhibits the BCR cell survival pathway leading to apoptosis. This represents the likelihood of tyrosine kinase inhibitors being effective targeted treatments for ZAP-70+ CLL cells.

The clinical course of chronic lymphocytic leukemia (CLL) is highly variable, and although some patients are treated at diagnosis, others may not require therapy for years.^1^ Biomarkers can help stratify these patients into indolent and aggressive disease categories. The aggressiveness of CLL is dependent on whether the leukemia cells have (60% of CLL population) or lack (40% of CLL population) mutations of the *immunoglobulin variable region of the heavy chain* (*IgV*_*H*_). Thus, patients with early-stage disease have a median survival of 8 years if they have unmutated *IgV*_*H*_ (Un-*IgV*_*H*_) and 24 years if they have mutated *IgV*_*H*_ (Mu-*IgV*_*H*_) disease.^2^ A surrogate marker for *IgV*_*H*_ mutational status is the expression of zeta-chain-associated protein 70 (ZAP-70); *IgV*_*H*_ mutated CLL cells are frequently ZAP-70 negative, whereas *IgV*_*H*_ unmutated cells are more typically ZAP-70 positive.^3^ ZAP-70 staining in CLL is not an all-or-nothing phenomenon, and to maximize the correlation with *IgV*_*H*_ mutational status, a ZAP-70-positive case is defined as ≥20% of the CLL cells staining for ZAP-70. Like *IgV*_*H*_ status, overexpression of ZAP-70 in CLL cells is associated with aggressive disease; time to treatment is 2.6 years for ZAP-70+ patients compared with 8 years for ZAP-70− patients independent of Rai stage.^3^ Thus, ZAP-70 is a rationale target for therapy in CLL.

Although the clinical relevance of ZAP-70 in CLL is well known, its molecular function is less understood. ZAP-70 is a member of the Syk family of protein tyrosine kinases and is normally involved in signal transduction of the T-cell receptor in T cells. ZAP-70 overexpression in malignant B cells, such as CLL cells, enhances the B-cell receptor (BCR) pathway. This pathway is a key mechanism for cell survival in CLL.^4,5^ Upon activation of the BCR, tyrosine kinase Lyn phosphorylates and activates Syk, leading to activation of downstream signaling pathways and upregulation of anti-apoptotic proteins, such as Mcl-1. CLL cells with both Un-*IgV*_*H*_ and high ZAP-70 expression show increased activation of proteins downstream of the BCR such as Akt, mitogen-activated protein kinase (MAPK), and NF-*κ*B.^4,6,7^ This suggests that alterations in the BCR signaling pathway through increased expression of the tyrosine kinase ZAP-70 are important in CLL disease progression.

Gefitinib is a tyrosine kinase inhibitor known for targeting the epidermal growth factor receptor (EGFR) and is used in the treatment of non-small-cell lung cancer and other cancers of epithelial origin.^8^ The drug is well tolerated, with rash and diarrhea being the only dose-limiting toxicities. Importantly to leukemias, it is not myelosuppressive.^9^ Apart from its effects on EGFR activity, gefitinib has shown activity against >20 other kinase targets, including Lyn and Syk.^10,11^ Gefitinib has been shown activity in acute myeloid leukemia (AML), myelodysplastic syndrome (MDS), and acute lymphocytic leukemia (ALL), inducing both differentiation and cell death *in vitro*.^12^ These effects are associated with inhibition of Syk phosphorylation. Thus, although gefitinib is used to treat lung cancer by inhibiting EGFR, it has potential utility in the treatment of CLL patients with high expression of Syk family members that include ZAP-70.

In this study we show that gefitinib selectively induces apoptosis in ZAP-70-expressing CLL cells, both when unstimulated and BCR activated. These effects are associated in both cases with a reduction in overall tyrosine phosphorylation and specific decreases in Lyn/Lck, Syk/ZAP-70, ERK1/2, and Akt phosphorylation. These changes produce a decreased expression of Mcl-1 and blocked anti-apoptotic signaling. Forced overexpression of ZAP-70 by lentiviral infection in the Raji B-cell line increases the sensitivity of the cells to gefitinib-induced apoptosis. However, normal T cells from CLL patients, which also express ZAP-70, are not affected by gefitinib. These results suggest that tyrosine kinase inhibitors such as gefitinib are a viable treatment option for ZAP-70+ CLL patients.

## Results

### Gefitinib targets ZAP-70+ CLL cells and leukemia cell lines

**Figure 1:**
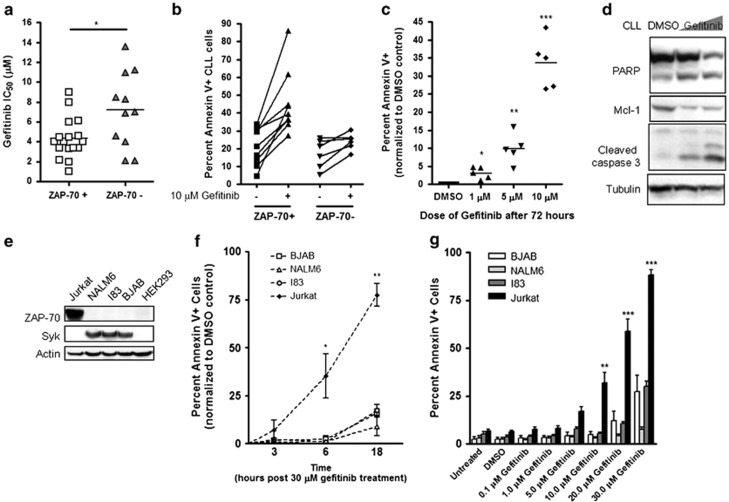
Gefitinib targets ZAP-70+ CLL cells. (**a**) Distribution of gefitinib IC_50_ values in ZAP-70+ (*n*=22 tested) or ZAP-70− (*n*=23 tested) patient samples after 24 h of treatment. Only values within tested range of 1–15 *μ*M are shown; values outside tested range were excluded. (**b**) Percentage of annexin V+ CLL cells from ZAP-70+ or ZAP-70− patients treated for 24 h with 10 *μ*M gefitinib. (**c**) Percentage of annexin V+ CLL cells from 5 different ZAP-70+ patients after 72 h of treatment with DMSO, and 1, 5, or 10 *μ*M gefitinib. (**d**) Lysates were collected after 24 h of CLL cells treated with DMSO, 10 *μ*M gefitinib, or 15 *μ*M gefitinib. (**e**) Western blot with lysates of ZAP-70− and ZAP-70+ lymphoid cell lines compared with ZAP-70− and Syk− HEK293 cell lines. (**f**) Treatment of lymphoid cell lines with 30 *μ*M gefitinib for 3, 6, and 18 h normalized to DMSO-treated control. Three independent experiments are shown with standard errors (paired *t*-test). (**g**) Lymphoid cell lines treated with 0.1 to 30.0 *μ*M gefitinib for 24 h. Cell death analyzed by staining with Annexin V-FITC and 7AAD and analysis by flow cytometry. Three independent experiments are shown with standard errors (paired *t*-test). *P<0.05, **P<0.01, or ***P<0.001

Given the efficacy of gefitinib in targeting Syk in AML,^11,12^ we investigated its efficacy in CLL cells expressing ZAP-70. CLL samples were defined as being ZAP-70+ if the number of positive cells was ≥20%. The concentration of drug required to reduce cell viability by 50% (IC_50_) for gefitinib in ZAP-70+ and ZAP-70− CLL cells was assessed using the MTT (3,3-(4,5-Dimethylthiazol-2-yl)-2,5-diphenyltetrazolium bromide) cell viability assay. The median gefitinb IC_50_ for ZAP-70+ CLL cells was 4.5 *μ*M and >15 *μ*M for ZAP-70− cells that was statistically significant (Table 1 and Figure 1a). Overall, 77% of ZAP-70+ patient cells and 30% of ZAP-70− patients responded to gefitinib treatment as defined by the median IC_50_ concentration. In addition, there did not appear to be cross-resistance to fludarabine in the ZAP-70+ samples, and most fludarabine-resistant cases were sensitive to gefitinib (Supplementary Table 1).

Interestingly, another EGFR tyrosine kinase inhibitor, erlotinib, had no activity against the ZAP-70+ CLL cells. There was also no significant difference between the median IC_50_ of fludarabine in ZAP-70+ and ZAP-70− CLL cells (5.4 *μ*M compared with 7.0 *μ*M). There was also no significant difference in the median IC_50_ values of gefitinib when all cases were stratified by *IgV*_*H*_ (7.0 *μ*M for Un-*IgV*_*H*_ compared with 8.3 *μ*M or Mu-*IgV*_*H*_) or if ZAP-70+ cases were stratified by mutational status (4.0 *μ*M for ZAP-70+/Mu-*IgV*_*H*_ compared with 6.0 *μ*M for ZAP-70+/Un-*IgV*_*H*_).

To confirm the MTT results, primary CLL cells were treated *in vitro* with gefitinib and cell death was analyzed by flow cytometry after 24 h. Although the median IC_50_ was 4.5 *μ*M by MTT assay 72 h post treatment, a higher dose of 10 *μ*M was chosen for cell death experiments because of the shorter time frame of 24 h to detect cell death. Gefitinib treatment increased apoptosis in ZAP-70+ primary CLL cells as detected by an increase in annexin V-stained cells. After 24 h, the number of annexin V-positive CLL cells ranged from 25 to 85% (Figure 1b). After 72 h, cell death was seen at doses as low as 1 *μ*M and increased in a dose-dependent manner (Figure 1c). Cell death was accompanied by increased cleavage of poly (ADP-ribose) polymerase (PARP) and caspase 3 and decreased Mcl-1 expression (Figure 1d and Supplementary Figure 1).

To test whether gefitinib could be give a synergistic apoptotic response with standard chemotherapeutic agents used to treat CLL, gefitinib was added alone or in combination with fludarabine. The combined effect of the two drugs failed to give a synergistic apoptotic response (Supplementary Figure 2a).

Based on standard definitions, ZAP-70+ CLL samples were defined as having ≥20% of their leukemia cells expressing ZAP-70,^3^ but changing the threshold of positive cells did not influence the results. To determine whether gefitinib was targeting only the ZAP-70+ cells or was affecting all cell populations, the viability of both ZAP-70+ and ZAP-70− cells was measured within the same sample. Gefitinib decreased the percentage of viable annexin V-ZAP-70+ CLL cells compared with dimethyl sulfoxide (DMSO)-treated controls in six different CLL samples (Supplementary Figure 2b). This indicates gefitinib is selectively inducing apoptosis in ZAP-70+ CLL cells. This preference for ZAP-70+ CLL cells cannot be from another gefitinib target, as CLL cells do not express EGFR and do not differentially express receptor-interacting protein kinase 2 (RIP2) or cyclin G-associated kinase 1 (GAK1; Supplementary Figure 3).

To support the role that ZAP-70 expression plays in gefitinib-induced apoptosis, we examined four leukemia/lymphoma cell lines for their sensitivity to gefitinib treatment. The B cell-derived NALM6, BJAB, and I-83 cell lines do not express ZAP-70, whereas the T cell-derived Jurkat cell line does express high levels of ZAP-70. Conversely, the B-cell lines expressed Syk, whereas Jurkat cells do not (Figure 1e). Human epithelial kidney cell line HEK293 was used as a negative control for both Syk and ZAP-70 expression. Gefitinib treatment over an 18-h time course revealed that only Jurkat cells showed significant apoptosis as detected by annexin V staining assay (Figure 1f). When the cell lines were treated with a range of gefitinib concentrations (0.1 to 30 *μ*M) only Jurkat cells showed a significant increase in apoptosis; there were 80% annexin V+ Jurkat cells but <40% annexin V+ B-cell lines after treatment of 30 *μ*M gefitinib for 18–24 h (Figures 1f and g). The Src tyrosine kinase inhibitor dasatinib did not show this bias toward Jurkat cells (Supplementary Figure 4), but this cannot rule out differential internalization of gefitinib in different cell lines.

**Figure 2:**
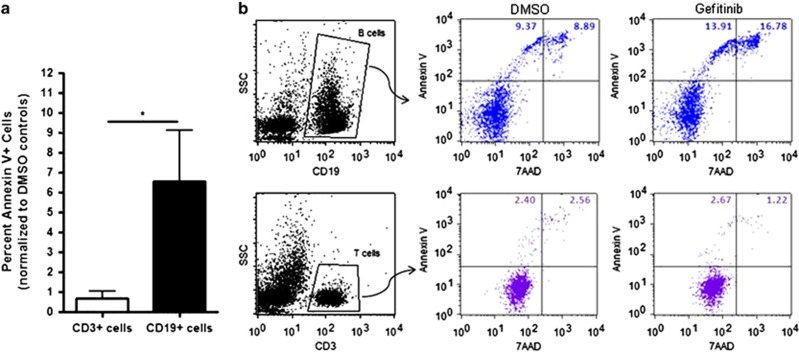
Gefitinib targets ZAP-70+ CLL cells but not ZAP-70+ T cells. (**a**) Percent Annexin V+ T cells and CLL cells from 4 different patients treated with 10 *μ*M gefitinib for 24 h as analyzed by flow cytometry. Graphed with standard errors and analyzed by Mann–Whitney test. (**b**) Flow cytometry plots of Annexin V-FITC and 7AAD+ CLL cells (gated as CD19-APC+ cells) or T cells (gated as CD3-PE+). *P<0.05

As gefitinib had a significant effect against the Jurkat leukemia T-cell line, we determined whether normal T cells were also sensitive to gefitinib. We isolated mononuclear cells from peripheral blood of ZAP-70+ patients with lymphocyte count <40 × 10^9^ cells/l and treated the cells with 10 *μ*M gefitinib. This dose was chosen to agree with gefitinib treatments on CLL cells. The T and B cells were kept at the ratio observed in the peripheral blood. The CLL cells showed apoptosis following treatment with gefitinib, whereas the T cells were resistant (Figures 2a and b). Even after 72 h of treatment with 10 *μ*M gefitinib, only ZAP-70+ CLL cells responded to treatment (Supplementary Figure 5), suggesting that gefitinib only targets malignant leukemia cells expressing ZAP-70.

### Gefitinib inhibits basal and BCR signaling, preventing downstream ERK and Akt activation

**Figure 3:**
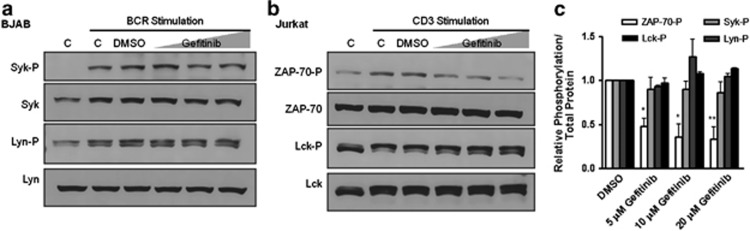
Gefitinib decreases ZAP-70 phosphorylation in Jurkat cells. (**a**) BJAB or (**b**) Jurkat cells treated with DMSO, and 5, 10, and 20 *μ*M gefitinib, and stimulated with anti-IgM or anti-CD3, respectively. Untreated control cells (C) were left unstimulated (lane 1) or stimulated (lane 2). Lysates collected after 1 h and analyzed by western blot for Syk/ZAP-70 and Lyn/Lck phosphorylation. (**c**) Bands quantified using ImageJ of three independent experiments. Standard errors are shown. *P<0.05, **P<0.01

In cell lines, gefitinib treatment reduced tyrosine phosphorylation of ZAP-70 in a dose-dependent manner in CD3-stimulated Jurkat T cells, but not Syk in BCR-stimulated BJAB B cells. There was no decrease in Lyn or Lck phosphorylation in BJAB or Jurkat cells, respectively (Figure 3).

**Figure 4:**
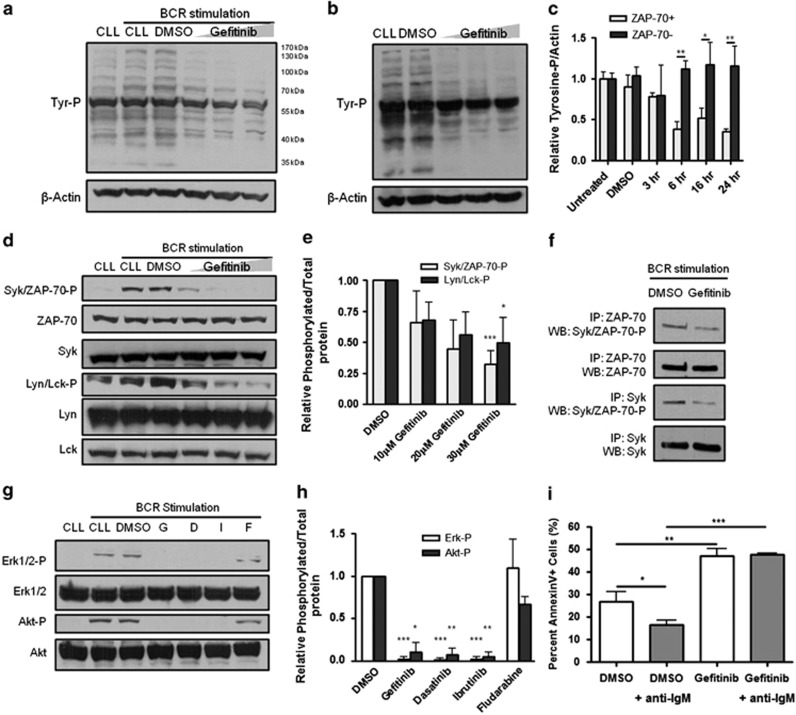
Gefitinib targets BCR pathway and inhibits survival response. Primary ZAP-70+ CLL cells were treated with DMSO, and 10, 20, or 30 *μ*M gefitinib, and stimulated with anti-IgM (**a**) or left unstimulated (**b**). Lysates were collected after 1 h and analyzed for tyrosine phosphorylation by western blot. The experiment was repeated with three different patient samples. (**c**) Lysates of a ZAP-70+ and a ZAP-70− CLL patient sample treated with 10 *μ*M gefitinib for 3, 6, 16, and 24 h analyzed by slot blot for tyrosine phosphorylation (unpaired *t*-test). (**d**) ZAP-70+ CLL cells were treated with DMSO, and 10, 20, or 30 *μ*M gefitinib, and stimulated with anti-IgM. Lysates were collected after 1 h and analyzed for ZAP-70/Syk and Lyn/Lck phosphorylation by western blot. The experiment was repeated with four different ZAP-70+ CLL samples, all of which were compared using densitometry (**e**) calculated using ImageJ. Densitometry values of phosphorylated Syk/ZAP-70 or Lyn/Lck first normalized to DMSO, and then to total protein. (**f**) Immunoprecipitation of ZAP-70 and Syk from a primary CLL patient sample and analyzed for tyrosine phosphorylation by western blot after 1 h of 30 *μ*M gefitinib treatment. Representative of *n*=3 is quantified in Supplementary Figure 6. (**g**) ZAP-70+ CLL cells were treated with DMSO, 30 *μ*M gefitinib (lane G), 10 *μ*M dasatinib (lane D), 10 *μ*M ibrutinib (lane I), or 10 *μ*M fludarabine (lane F) and stimulated with anti-IgM. The experiment was repeated in two different ZAP-70+ CLL samples and compared using densitometry (**h**). Lysates were collected after 1 h and analyzed for ERK1/2 and Akt phosphorylation by western blot. The experiment was repeated with two different patient samples. (**i**) ZAP-70+ CLL sample was pretreated with 10 *μ*M gefitinib for 30 min, stimulated with plate-immobilized anti-IgM for 30 min, and cultured for 24 h. Samples were stained with Annexin V-FITC and analyzed by flow cytometry (unpaired *t*-test). Single representative experiment done in triplicate with S.D. is shown. The experiment repeated with three different patient samples. *P<0.05, **P<0.01, or ***P<0.001

In primary CLL cells, we found gefitinib treatment reduced total cellular tyrosine phosphorylation, both from the basal level and from the BCR-stimulated level, in ZAP-70+ CLL cells over a range of gefitinib concentrations and incubation times (Figures 4a–c). However, this was not observed in ZAP-70− CLL cells. The decrease in overall cellular tyrosine phosphorylation was evident after 1 h, and decreased further after 24 h (Figures 4a–c). Tyrosine phosphorylation did not decrease in ZAP-70− CLL cells treated with increasing doses of gefitinib (Supplementary Figure 6a). However, even at doses as low as 1 *μ*M, gefitinib decreased tyrosine phosphorylation in ZAP-70+ CLL cells (Supplementary Figure 6c). As a control, erlotinib failed to decrease tyrosine phosphorylation in primary CLL cells (data not shown). Unlike cell lines, there were specific decreases in both Syk/ZAP-70 and Lyn/Lck phosphorylation in ZAP-70+ CLL cells after 1 h of gefitinib treatment (Figure 4d). Quantification of these decreases showed that Syk/ZAP-70 phosphorylation appeared to decrease slightly more than Lyn/Lck phosphorylation after gefitinib treatment (Figure 4e).

Because of the fact that phospho-antibodies recognized both Syk and ZAP-70 phosphorylation, we performed immunoprecipitation using specific Syk and ZAP-70 antibodies and western blotting for phospho-tyrosine after BCR stimulation. Each approach showed that both Syk and ZAP-70 phosphorylation decreased following gefitinib treatment (Figure 4f and Supplementary Figure 6d). Immunoprecipitation was also performed with phospho-tyrosine antibodies and then blotted first for ZAP-70 and then for Syk (data not shown).

The activity of gefitinib was compared with ibrutinib and dasatinib, the two tyrosine kinase inhibitors under clinical investigation in CLL. The downstream effect of gefitinib was the same as these other two drugs and all three prevented phosphorylation of Akt and ERK after BCR stimulation (Figures 4g and h). Even when the primary CLL cells had high basal Akt phosphorylation, which did not increase with BCR stimulation, there was still an inhibition of phosphorylation with the tyrosine kinase inhibitors (data not shown). This inhibition was not observed with fludarabine that was used as a negative control. BCR stimulation alone served as a positive control.

As signaling through the BCR promotes cell survival, we determined whether inhibition of this signaling pathway by gefitinib decreased survival. Primary CLL cells were treated with 10 *μ*M gefitinib, BCR activated for 30 min by immobilized anti-IgM, and cell death quantitated by annexin-V staining after 24 h. We found that anti-IgM protected CLL cells from spontaneous apoptosis but failed to protect CLL cells from gefitinib treatment (Figure 4i).

### Forced overexpression of ZAP-70 increases sensitivity to gefitinib

**Figure 5:**
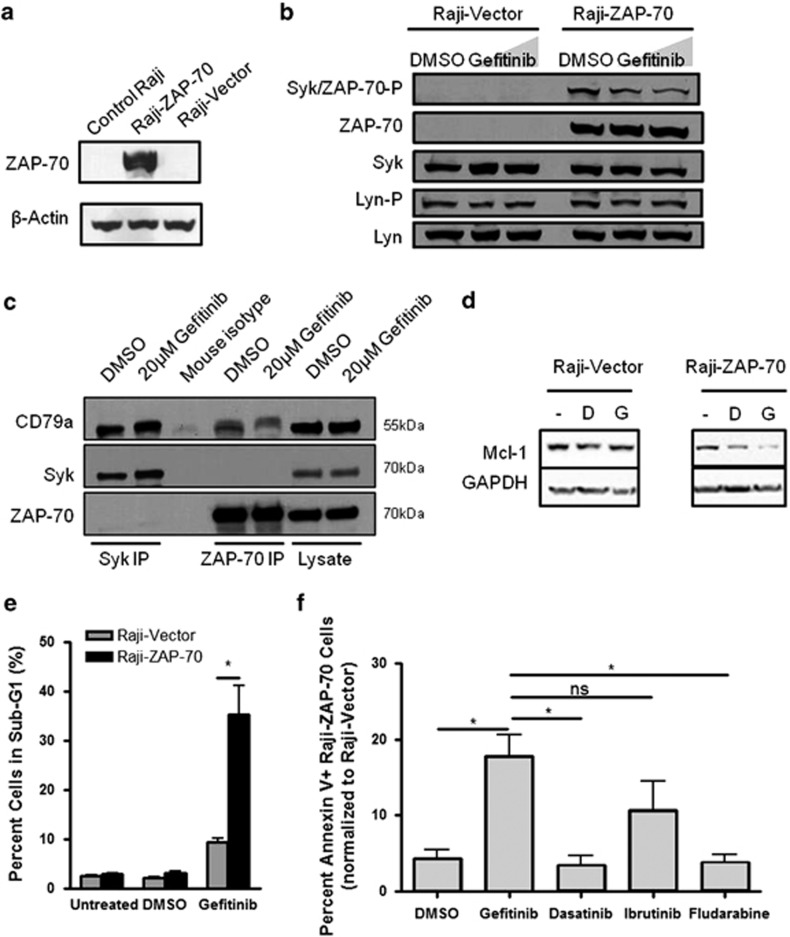
ZAP-70 expression sensitizes Raji cells to gefitinib treatment. Raji cells transduced with GFP control vector or ZAP-70 vector (**a**) were treated with DMSO, and 20 or 30 *μ*M gefitinib, or left untreated for 1 h and analyzed for Syk/ZAP-70 phosphorylation by western blot (**b**). (**c**) Co-immunoprecipitation of Syk or ZAP-70 with CD79a in Raji-ZAP-70 cells treated with DMSO or 20 *μ*M gefitinib for 24 h and then lysed in 0.2% CHAPS buffer. (**d**) Lysates of untreated (-) and treated with DMSO (D) or gefitinib (G) Raji-ZAP-70 and Vector cells were analyzed by western blot for Mcl-1 degradation. (**e**) Raji-vector and Raji-ZAP-70 cells were treated with 20 *μ*M gefitinib and stained with propidium iodide and analyzed by flow cytometry. Results from three independent experiments are shown (unpaired *t*-test). (**f**) Raji-vector and Raji-ZAP-70 cells were treated with DMSO, 20 *μ*M gefitinib, 30 *μ*M dasatinib, 20 *μ*M ibrutinib, or 20 *μ*M fludarabine for 24 h and then stained with Annexin V-APC and 7-AAD and analyzed by flow cytometry. Cell death of Raji-ZAP-70 cells normalized to Raji-vector cells. Results from three independent experiments are shown. *P<0.05

To determine whether ZAP-70 plays a role in the susceptibility of a patient to gefitinib, we tested the response of the ZAP-70-negative lymphoma-derived B-cell line Raji transduced with GFP-expressing vector or ZAP-70-expressing vector. Expression of ZAP-70 was confirmed by western blot (Figure 5a) and flow cytometry and expression of GFP was confirmed by flow cytometry (Supplementary Figure 7). We found that gefitinib treatment lowered Syk/ZAP-70 phosphorylation but not Lyn phosphorylation in ZAP-70-expressing Raji cells (Figure 5b). Western blot analysis showed constitutive phosphorylation of Syk/ZAP-70 in Raji cells overexpressing ZAP-70 (Figure 5b), which is congruent with previously published literature.^13^ Upon BCR activation, Syk/ZAP-70 binds to the tyrosine phosphorylated co-receptor CD79a. We found that there was no difference in Syk or ZAP-70 binding to CD79a after gefitinib treatment (Figure 5c). This suggests that CD79a is still tyrosine phosphorylated after gefitinib treatment.

We next investigated the sensitivity of gefitinib in ZAP-70-overexpressing Raji cells. Raji cells overexpressing ZAP-70 had increased sensitivity to gefitinib compared with cells with vector alone as measured by greater degradation of Mcl-1 (Figure 5d), greater DNA fragmentation as measured by sub-G1 peak analysis (Figure 5e), and greater annexin V and 7-amino-actinomycin D (7AAD) staining (Figure 5f). These results were confirmed with different cell passages, and different cell death staining techniques (PI staining and annexin V staining). We further knocked down ZAP-70 in Raji cells and showed reduced Erk phosphorylation and Mcl-1 expression, indicating changes in downstream targets of gefitinib were due to ZAP-70 overexpression (Supplementary Figure 8). In addition, gefitinib treatment was compared with dasatinib and ibrutinib. Gefitinib had the greatest effect of all the drugs on Raji cells overexpressing ZAP-70, as compared with Raji cells with vector alone (Figure 5f). Dasatinib had little effect on Raji cells, whether they overexpressed ZAP-70 or not, only increasing cell death by 3–10%. Ibrutinib had a greater effect on ZAP-70-overexpressing Raji cells, as compared with the non-ZAP-70-expressing cells, but there was less cell death than seen with gefitinib using this same drug dosage (20 *μ*M). This increased sensitivity of the ZAP-70-expressing Raji cells was not seen with fludarabine treatment, and this was used as a negative control (Figure 5f).

Unfortunately, the complementary experiments to treat Jurkat cells with ZAP-70 knockdown were not feasible because knockdown of ZAP-70 led to increased cell death compared with control siRNA (Supplementary Figure 9).

## Discussion

In this study the tyrosine kinase inhibitor gefitinib, originally used to inhibit EGFR kinase activation in lung cancer, is also cytotoxic to primary CLL cells that overexpress ZAP-70. When these cells undergo BCR activation, gefitinib can inhibit phosphorylation of Lyn/Lck, Syk/ZAP-70, ERK1/2, and Akt within 1 h. These results are similar to those seen with 5–10 *μ*M gefitinib in AML and MDS cells,^11^ where gefitinib functions through an EGFR-independent mechanism targeting Syk activation. Using the MTT assay, which we and others have shown to be predictive of clinical response to fludarabine and chlorambucil,^14^ the median IC_50_ of gefitinib was 4.5 *μ*M in ZAP-70+ CLL cells but >15 *μ*M in ZAP-70− cells.

There has been considerable interest in the evaluation of tyrosine kinase inhibitors for the treatment of CLL. Dasatinib is normally used to treat chronic myeloid leukemia and is a tyrosine kinase that inhibits the Src family member Abl.^15^ However, it is also cytotoxic to CLL cells, including ZAP-70+ cells, and it has been suggested that dasatinib is targeting tyrosine kinase Lyn.^15^ In addition, Syk has been targeted in CLL with the tyrosine kinase inhibitor, R406 (fostamatinib). The effect of R406 was greatest in cells with high levels of Syk that were Un-*IgV*_*H*_ and expressed ZAP-70.^16^ However, R406 had no effect on the phosphorylation of other tyrosine kinases, such as ZAP-70.^16^ Recent evidence has indicated that these findings are clinically relevant as the pro-drug for R406, fostamatinib disodium (FosD), is clinically active in CLL patients.^17^ Two novel Syk inhibitors, PRT318 and P505-15, have recently been shown to suppress CLL activation and migration *in vitro*.^18^ Besides these tyrosine kinase inhibitors, the Bruton's tyrosine kinase inhibitor, ibrutinib, has shown potent activity in CLL, both *in vitro* and *in vivo*,^19,20^ and the multikinase inhibitor, sorafenib, has also been shown to induce apoptosis in CLL cells *in vitro*.^6^ These inhibitors were active against both ZAP-70– and ZAP-70+ CLL cells, whereas gefitinib was the only tyrosine kinase inhibitor shown to selectively sensitize ZAP-70+ CLL cells to undergo apoptosis. The long-term toxicity and efficacy of these tyrosine kinase inhibitors in CLL is unknown and it will be years before they become FDA approved for use in this disease. In contrast, gefitinib is already approved by the FDA for the treatment of lung cancer and has been used for many years in the clinic. It has the potential advantage in CLL in being specifically cytotoxic to ZAP-70+ CLL cells, and not being myelo- or immuno-suppressive.

Gefitinib has recently been shown to accumulate in solid tumors. Haura *et al.*^21^ found 22 *μ*M in lung tumor, and McKillop *et al.*^22^ found 16.7 *μ*M in breast tumor. These doses were 40 and 42 times higher than the concentration observed in the plasma, respectively. We predict that this accumulation of gefitinib at the cancer site would also occur in leukemia patients. Therefore, low plasma concentrations from patients with solid tumors may not be appropriate values to consider when testing doses of gefitinib on leukemic cells that reside in the blood and lymphoid tissues. In addition, *in vitro* experiments cannot recapitulate the dosing scheme that would be used *in vivo*. Gefitinib cytotoxic concentrations in ZAP-70+ CLL cells were similar to its concentrations that induce apoptosis in AML cells. In the future, we will focus on *in vivo* models testing gefitinib in various drug combinations for effectiveness.

The blood and lymphatic systems consist of distinct microenvironments that include blood, bone marrow, spleen, and lymph nodes. As cells traffic through these microenvironments, dynamic cell–cell interactions occur between mobile cells and tissue-resident cells. ZAP-70+ CLL cells tend to localize to the nodes and this is associated with more aggressive disease.^3^ One of the most important signals from the microenvironment for cell survival is BCR activation.^5,23,24^ Upon activation of the BCR, the tyrosine kinase Lyn phosphorylates and activates Syk, leading to activation of downstream signaling pathways such as Akt, MAPK, and NF-*κ*B, upregulation of anti-apoptotic proteins such as Mcl-1, and inactivation of pro-apoptotic protein BIM. These changes lead to increased cell survival.^24,25^ CLL cells with both Un-*IgV*_*H*_ and high ZAP-70 expression show increased BCR signaling.^24,25^ This suggests that alterations in the BCR signaling pathway are important in CLL disease progression. In the present study, we showed that gefitinib blocked both ERK and Akt activation leading to a decrease in Mcl-1 expression and apoptosis. This mechanism of cell death may be common among the tyrosine kinase inhibitors.^26^ The evidence that ZAP-70 expression sensitizes cells to gefitinib and that gefitinib targets the BCR pathway both indicate that this drug may have activity in the microenvironment. In particular, gefitinib may have an effect in the lymph node microenvironments where BCR signaling occurs^27^ and ZAP-70 expression is upregulated.^28^ It is important to note that the complexity of feedback loops and interactions of ZAP-70 in CLL cells are not clearly understood, making it difficult to definitively determine the precise action of gefitinib. This will be the focus of future investigations.

Despite inefficient tyrosine kinase activity in CLL,^29^ ZAP-70 still plays an important role in the overactivation of the BCR pathway. Although the kinase domain is not required for enhanced signaling, inhibition of its kinase activity may cause steric hindrance or prevent conformational changes of signaling complexes preventing downstream signaling events.

Overall, gefitinib selectively targets CLL cells expressing ZAP-70. This indicates that tyrosine kinase inhibitors could be used to selectively treat patients with high ZAP-70-expressing CLL cells. As gefitinib is already in clinical use in lung cancer patients, and lacks suppression of the bone marrow or immune system, further studies are warranted to investigate the clinical activity of gefitinib in ZAP-70+ CLL patients.

## Materials and Methods

### Cell isolation and culture

Peripheral blood samples were collected from patients following informed consent in accordance with the Research Ethics Board at the University of Manitoba. Samples were mixed with RosetteSep (Stemcell Technologies, Vancouver, BC, Canada) if the lymphocyte count was <40 × 10^9^/l and then purified on a Ficoll-Paque gradient (GE Healthcare, Cleveland, OH, USA). Red blood cells (RBCs) were lysed with a RBC lysis buffer (eBioscience, San Diego, CA, USA). All blood samples were processed within 24 h after collection and used fresh. For experiments, the leukemia cells were grown in Hybridoma serum-free medium (SFM, Life Technologies, Carlsbad, CA, USA).

BJAB (ATCC, Burlington, ON, Canada), NALM6 (DSMZ, Braunschweig, Germany), I83 (kind gift from Dr. Panasci, McGill University, Montreal, Canada), Jurkat (ATCC), and Raji+/− ZAP-70 (kind gifts from Dr. Marshall, University of Manitoba, Winnipeg, MB, Canada) cell lines were all cultured in Hyclone RPMI with 10% Hyclone fetal bovine serum (FBS; Thermo Fisher Scientific, Waltham, MA, USA).

### Drugs and stimuli

Gefitinib (LC Laboratories, Woburn, MA, USA), erlotinib (LC Laboratories), dasatinib (LC Laboratories), ibrutinib (SelleckBio, Houston, TX, USA), and fludarabine (Sigma, St. Louis, MO, USA) were all dissolved in DMSO (Thermo Fisher Scientific) and added. B cells were stimulated with 10 *μ*g/ml biotinylated Fab_2_'IgM (Southern Biotech, Birmingham, AL, USA) and T cells were stimulated with 2 *μ*g/ml LEAF-purified anti-CD3 (BioLegend, San Diego, CA, USA). For western blot experiments, 10 *μ*g/ml soluble anti-IgM was added and cells were lysed within 1 h. For cell death analysis after 24 h, 0.1 *μ*g anti-IgM was immobilized in Hanks' buffered salt solution (HBSS, Life Technologies) in a 96-well Falcon plate (BD, Franklin Lakes, NJ, USA) overnight, and then washed before addition of CLL cells.

### Cell viability assays

For MTT assays, 3 × 10^7^ CLL cells in 3 ml of Hybridoma SFM were added to tubes (Sarstedt, Nümbrecht, Germany) and treated with 1, 2, 5, 10, or 15 *μ*M of the drug for 24 h. Cells were washed in HBSS and seeded into 96-well plates for 3 days. On day 3, MTT (Sigma) was added to a final concentration of 0.25 mg/ml. Plates were incubated for 5 h at 37 °C with 5% CO_2_, and absorbance was measured at a wavelength of 540 nm.

For flow cytometry, samples were collected, washed with 1 × annexin V Binding Buffer (BD Biosciences, Franklin Lakes, NJ, USA), and then stained with 7AAD (BD) and annexin V-fluorescein isothiocyanate (FITC; BD) or annexin V-allophycocyanin (APC; BD). Transduced Raji cells were stained instead with propidium iodide (PI) or annexin V-APC (BD) because of the GFP vector. Samples were examined using a BD FACSCalibur.

### Western blotting and immunoprecipitation

Cell lysates were collected at the indicated times in 1% NP-40 lysis buffer with complete protease inhibitor tablet (Roche, Basel, Switzerland), 1 mM phenylmethanesulfonylfluoride (PMSF), and 2 mM sodium orthovanadate (New England BioLabs, Ipswich, MA, USA). Protein levels were quantified with a Pierce BCA kit (Thermo Fisher Scientific) according to the manufacturer's instructions. Samples were run on 8–10% polyacrylamide gels and transferred onto nitrocellulose membranes (Bio-Rad, Hercules, CA, USA) blocked in 5% BSA (Sigma) or milk in TBS-T as per the antibody manufacturer's suggestions. Primary antibodies included rabbit or mouse anti-ZAP-70 (Cell Signaling, Beverly, MA, USA), rabbit anti-Syk/ZAP-70-P (Cell Signaling), rabbit anti-Lyn (Cell Signaling), rabbit anti-Lyn-P (Epitomics, Burlingame, CA, USA), mouse anti-Lck (Cell Signaling), rabbit anti-Btk (Cell Signaling), rabbit anti-Btk-P (Cell Signaling), rabbit anti-ERK1/2 (Cell Signaling), rabbit or mouse anti-ERK1/2-P (Cell Signaling), rabbit anti-Akt (Cell Signaling), rabbit anti-Akt Ser-P (Cell Signaling), mouse anti-tyrosine-P (Millipore, Darmstadt, Germany), rabbit anti-Mcl-1 (Cell Signaling), rabbit anti-PARP (Cell Signaling), rabbit anti-cleaved caspase 3 (Cell Signaling), mouse anti-glyceraldehyde-3-phosphate dehydrogenase (anti-GAPDH; Sigma), rabbit anti-*α*-tubulin (Cell Signaling), and rabbit or mouse anti-*β-*actin (Sigma). Secondary antibodies were goat anti-rabbit-HRP or anti-mouse-HRP (Bio-Rad). Detection of protein was with Pierce ECL or Pierce Supersignal Pico (Thermo Fisher Scientific) reagents. Co-immunoprecipitation was carried out with 500 *μ*g of protein in 0.2% CHAPS lysis buffer containing 150 mM NaCl, 20 mM Tris, 10% glycerol, 2 mM EDTA, 1 mM PMSF, 2 mM sodium orthovanadate, and complete protease inhibitor tablet (Roche). Lysates were incubated at 4 °C with primary antibody mouse anti-Syk (Abcam, Cambridge, UK) or mouse anti-ZAP-70 (Cell Signaling) on a rotator overnight. Pierce protein G plus agarose bead slurry (Thermo Fisher Scientific) was added in a final dilution of 1 : 10 for 2 h and the procedure for western blot was followed. Entire immunoprecipitate supernatant was loaded on to a gel lane. Immunoprecipitations were done in the same way, with the exception of using 1% NP-40 lysis buffer.

### Flow cytometry

For extracellular staining of T and B cells, peripheral mononuclear cells were stained with annexin V-FITC, anti-CD3-PE, 7AAD, and anti-CD19-APC (BD Biosciences). Cells gated on either CD19+ or CD3+ were then analyzed for expressions of annexin V and 7AAD.

For intracellular staining of ZAP-70 in cell death experiments, RosetteSep and ficoll-purified CLL cells were first surfaced stained with annexin V-APC and 7AAD, then fixed with solution A (Beckman Coulter, Brea, CA, USA) for 12 min at 37 °C, washed with PBS, permeabilized with solution B (Beckman Coulter) for 5 min at room temperature, and then stained with mouse anti-human ZAP-70-FITC (Beckman Coulter) for 15 min. Mouse IgG1-FITC (BD) was used as an isotype control. Unstained and single-stained controls were always included in all flow cytometry experiments. All sample data were acquired on BD FACSCalibur and analyzed using CellQuest Pro software (BD). CLL cell samples were considered ZAP-70+ if ≥20% of the cells stained positively.

The ZAP-70 status of each patient sample was determined by diagnostic flow cytometry. Whole blood was stained with anti-CD19, anti-CD5, anti-CD38, and anti-ZAP-70. The negative control is a normal blood sample that should not have CD19+CD5+ leukemic cells. The positive control is the autologous CD19-CD5+ZAP-70+ T cells.

### Transduction of ZAP-70

Vectors encoding GFP pWPTS or vector encoding ZAP-70 pWPTS-ZAP-70 were packaged into lentivirus by co-transfection with pCMV-R8.91 and pMD.G into HEK-293 T cells.^30,31^ All vectors were kindly provided by Dr. Ferenc Boldizsar (University of Pécs, Pécs, Hungary). Raji cells (originally from DSMZ) were transduced with lentiviral particles using a spin protocol as previously described.^32,33^ Expression of ZAP-70 was confirmed by western blot and flow cytometry.

### Statistical analysis

Graphs were created and statistics were performed using GraphPad Prism4 software (GraphPad Software Inc., San Diego, CA, USA). Unless otherwise noted, a paired or unpaired two-tailed *t*-test was performed according to the nature of data. Statistical significance was noted in the figures as *P<0.05, **P<0.01, or ***P<0.001. Densitometry was calculated using ImageJ (Wayne Rasband; National Institute of Mental Health, Bethesda, MD, USA).

## Figures and Tables

**Table 1:** Gefitinib, erlotinib, and fludarabine IC_50_ values determined by MTT assays in ZAP-70+, ZAP-70−, mutated *IgV*_*H*_, and unmutated *IgV*_*H*_ primary CLL cells treated for 24 h

**CLL patients**	**Gefitinib median IC**_**50**_	**Erlotinib median IC**_**50**_	**Fludarabine median IC**_**50**_
ZAP-70+	4.5 *μ*M (*n*=22)^a^	>40.0 *μ*M (*n*=8)	5.4 *μ*M (*n*=14)
ZAP-70−	>15.0 *μ*M (*n*=23)	>40.0 *μ*M (*n*=10)	7.0 *μ*M (*n*=16)
Mutated (Mu-) *IgV*_*H*_	7.0 *μ*M (*n*=26)	>40.0 *μ*M (*n*=12)	7.0 *μ*M (*n*=23)
Unmutated (Un-) *IgV*_*H*_	8.3 *μ*M (*n*=13)	>40.0 *μ*M (*n*=5)	5.4 *μ*M (*n*=10)
ZAP-70+ and Mu-*IgV*_*H*_	4.0 *μ*M (*n*=9)	>40.0 *μ*M (*n*=2)	13.2 *μ*M (*n*=6)
ZAP-70+ and Un-*IgV*_*H*_	6.0 *μ*M (*n*=11)	>40.0 *μ*M (*n*=5)	5.4 *μ*M (*n*=8)

a Statistical significance of gefitinib median IC_50_ (*P*<0.05) between ZAP-70+ and ZAP-70− cells.

## Abbreviations

7AAD: 7-amino-actinomycin D
ALL: acute lymphocytic leukemia
AML: acute myeloid leukemia
APC: allophycocyanin
BCR: B-cell receptor
CLL: chronic lymphocytic leukemia
DMSO: dimethyl sulfoxide
EGFR: epidermal growth factor receptor
FBS: fetal bovine serum
FITC: fluorescein isothiocyanate
FosD: fostamatinib disodium
GAPDH: glyceraldehyde-3-phosphate dehydrogenase
GAK1: Cyclin *G Associated Kinase*
HBSS: Hanks' balanced salt solution
IC_50_: inhibitory concentration at 50%
Mu-*IgV*_*H*_: mutated *immunoglobulin variable region of heavy chain*
MAPK: mitogen-activated protein kinase
MDS: myelodysplastic syndrome
MTT: 3-(4,5-Dimethylthiazol-2-yl)-2,5-diphenyltetrazolium-bromide
PARP: poly (ADP-ribose) polymerase
PI: propidium iodide
PMSF: phenylmethanesulfonyl fluoride
RBC: red blood cell
RIP2: receptor-interacting protein kinase 2
Un-*IgV*_*H*_: unmutated *immunoglobulin variable region of heavy chain*
ZAP-70: zeta-chain-associated protein 70
