# Supplementary material for: Gefitinib targets ZAP-70-expressing chronic lymphocytic leukemia cells and inhibits B-cell receptor signaling
Source: Cell Death Dis. 2014 Oct 2;5(10):e1439–. doi: 10.1038/cddis.2014.391 (PMC4649506; doi:10.1038/cddis.2014.391)
Supplement: Supplementary Information [file cddis2014391x1.doc]

**Gefitinib targets ZAP-70+ chronic lymphocytic leukemia cells and inhibits B cell receptor signaling.**

**Supplementary Materials**

**Supplementary Table 1.** Clinical parameters (gender, age, Rai stage, lymphocyte count, and treatments), disease biomarkers (ZAP-70, CD38, and mutational status), and drug (gefitinib, erlotinib, and fludarabine) IC_50_ values (in μM) in all primary CLL patient samples used in MTT assays. Patients 1-22 are ZAP-70 positive, while patients 23-45 are ZAP-70 negative.


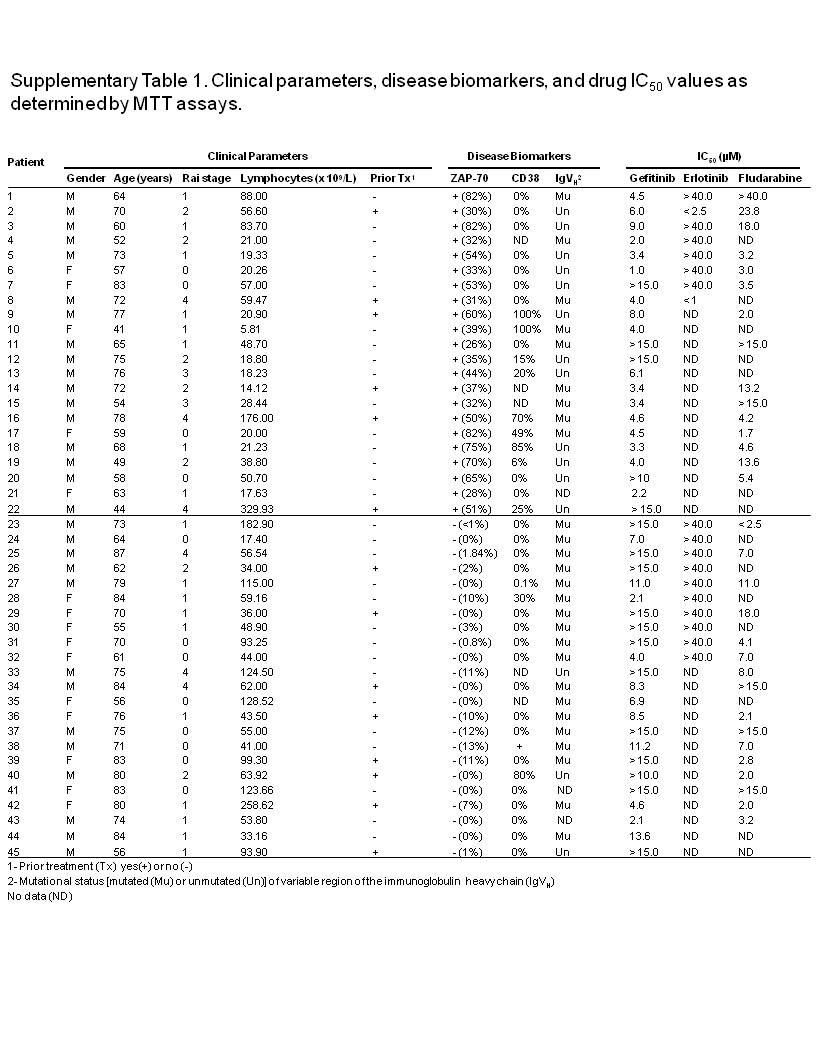


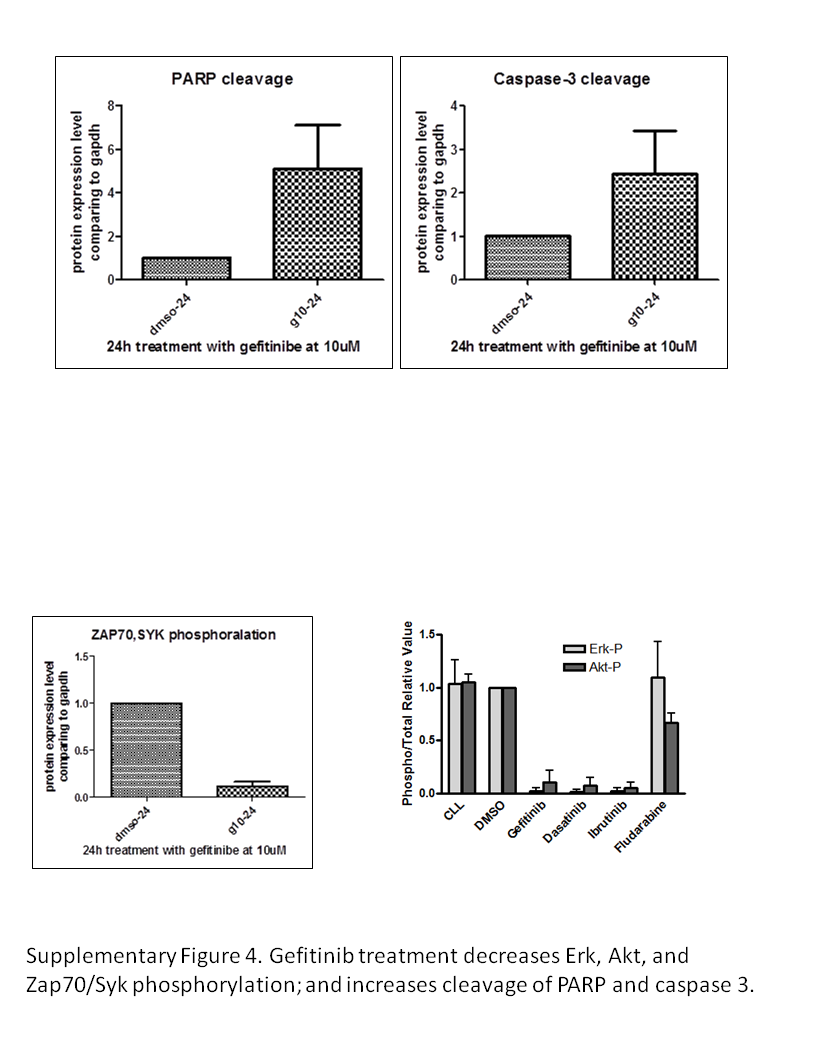

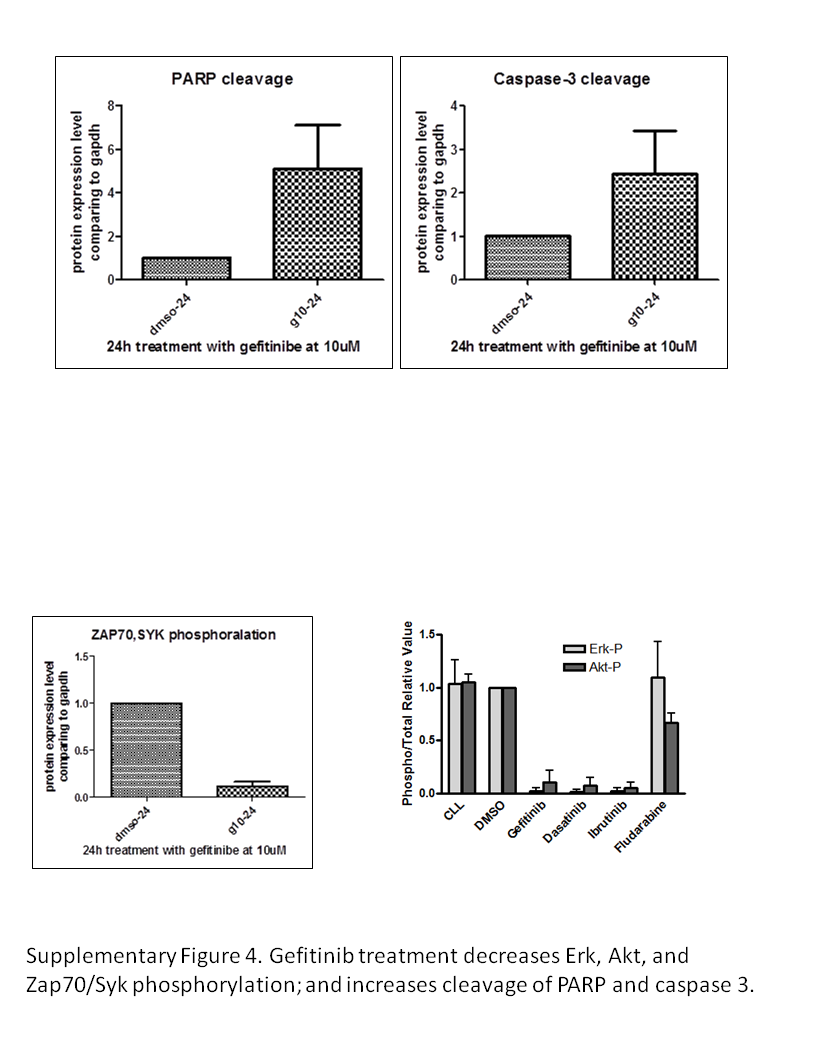


**Supplementary Figure 1. Gefitinib treatment of CLL cells increases cleavage of PARP and caspase 3**. Primary ZAP-70+ CLL cells were treated with DMSO or 10 μM gefitinib for 24 hours then lysed and analyzed by western blot. Cleavage products quantified using Image J. Results from at least 3 independent experiments are shown.

**a b**

**Supplementary Figure 2. Gefitinib decreases the percentage of viable ZAP-70+ CLL cells, but does not syngerize with fludarabine.** (a) Six different ZAP-70+ CLL patient samples (583, 607, 714, 840, 637, and 420) were treated with 10 μM fludarabine with or without 10 μM gefitinib for 24 hours, then stained with Annexin V-FITC and 7-AAD. (b) Percentage of annexin V- ZAP-70+ CLL cells from 6 different patients after 24 hour treatment with DMSO or 10 μM gefitinib (paired t test p≤0.001).Y-axis represents percentage of ZAP-70+ cells gated on Annexin V+ cells.

**
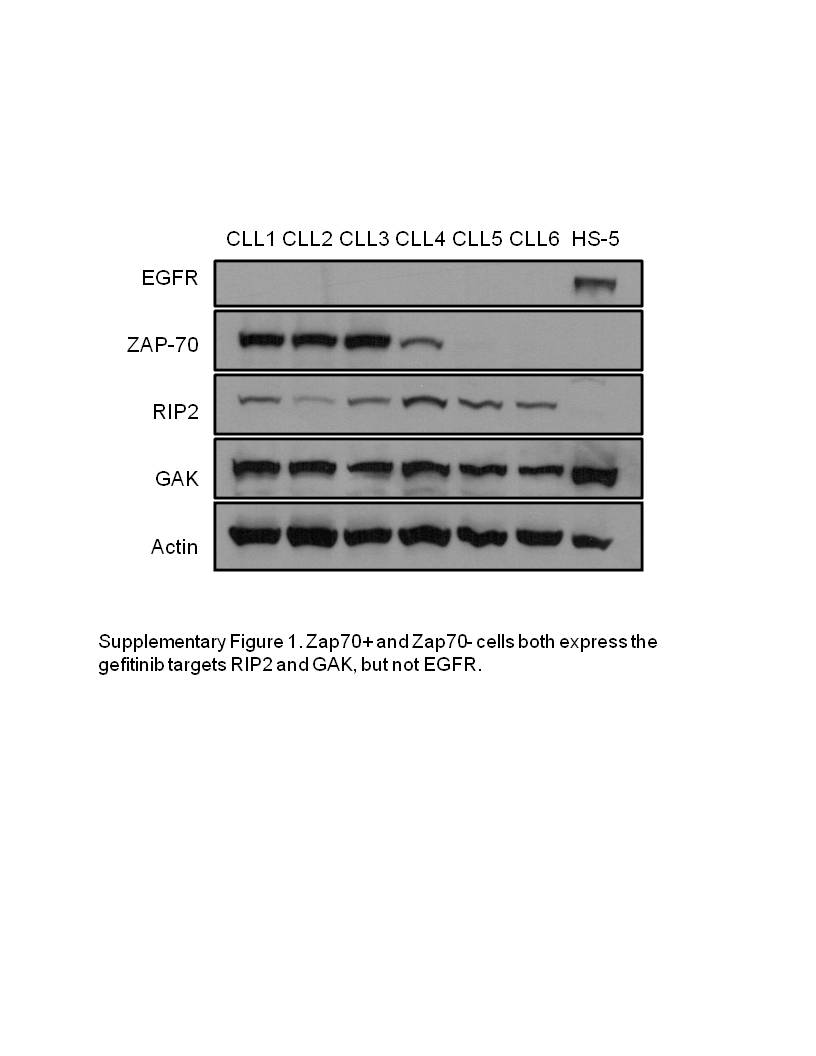
**

**Supplementary Figure 3. ZAP-70+ and ZAP-70- CLL cells do not express EGFR, and do not differentially express gefitinib targets RIP2 and GAK**. Lysates of primary CLL cells (CLL1-6) collected on freshly isolated samples. CLL1-3 are strongly ZAP-70+, CLL4 is moderately ZAP-70+, and CLL5-6 are ZAP-70-. HS-5 cell lysate used as positive control for EGFR and negative control for ZAP-70.


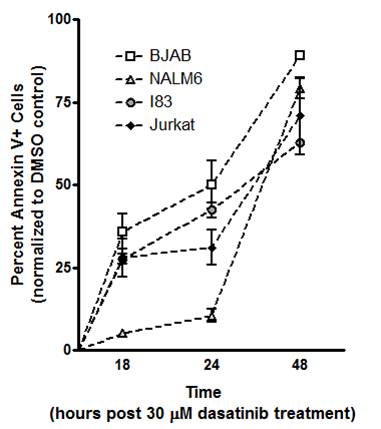


**Supplementary Figure 4. Dasatinib does not preferentially target ZAP-70+ Jurkat cells**. Treatment of lymphoid cell lines BJAB, Jurkat, NALM6, and I83 with 30 μM dasatinib for 18, 24, 48 hours normalized to DMSO-treated control. Cell death analyzed by Annexin V-FITC and 7AAD staining and flow cytometry analysis. Representative experiment shown with standard deviations. Three independent experiments performed.


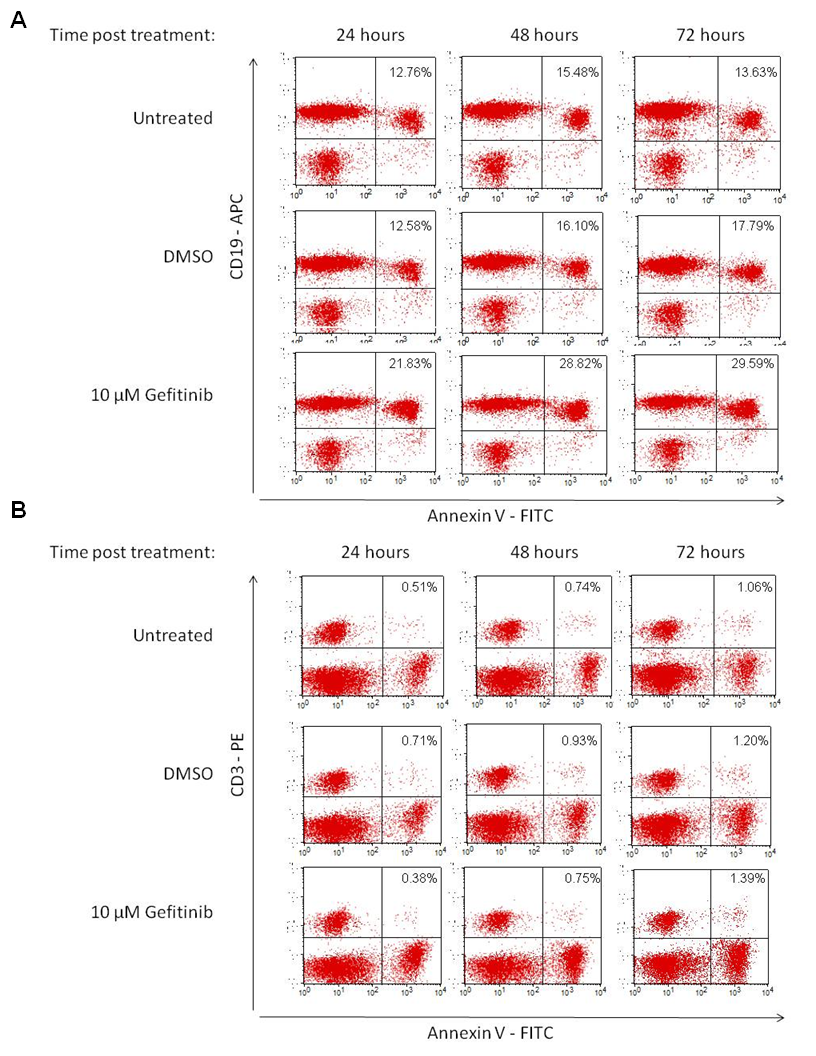


**a**

**b**

**Supplementary Figure 5. Gefitinib induces cell death of CLL B cells not T cells**. Peripheral mononuclear cells of a CLL patient with low count were isolated and treated with DMSO or 10 μM gefitinib for 24, 48, and 72 hours. Samples stained with Annexin V-FITC, anti-CD3-PE, 7AAD, and anti-CD19-APC and analyzed by flow cytometry using BD FACSCalibur. Results were confirmed using two analysis methods: gating on lymphocytes and comparing CD19 (a) or CD3 (b) versus Annexin V and also by gating on CD3 or CD19 and analyzing Annexin V versus 7AAD (Figure 3).


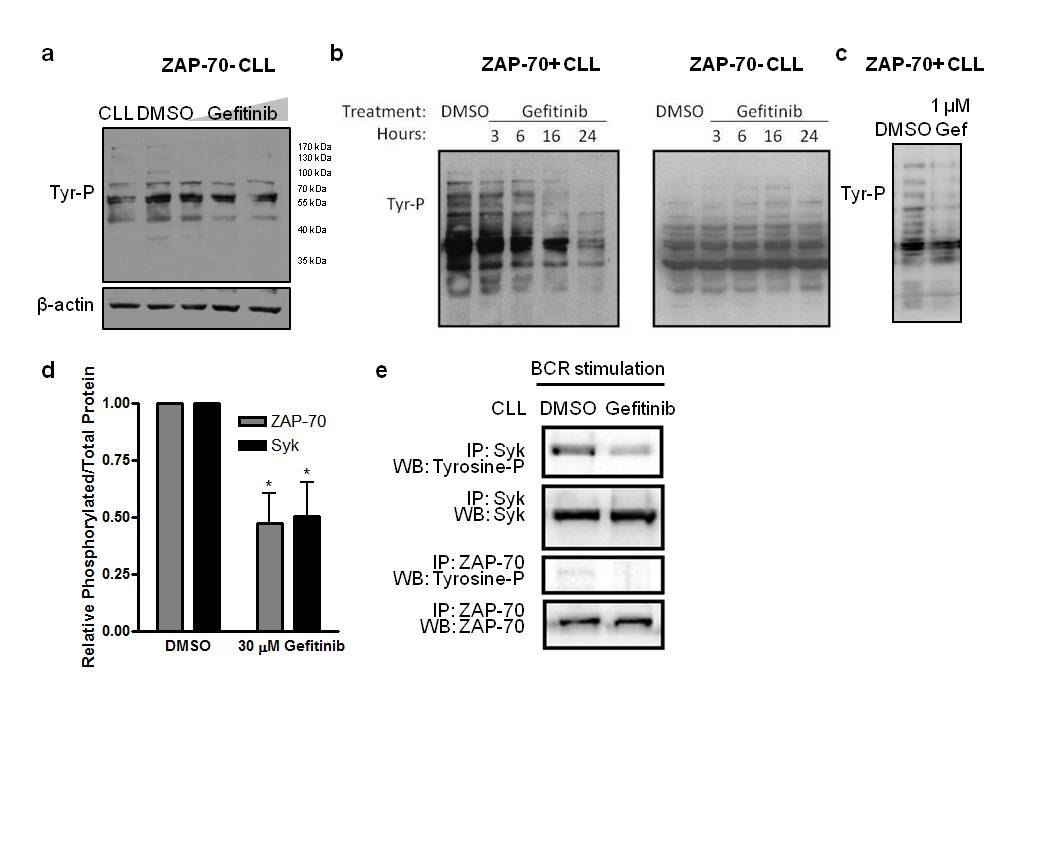


**Supplementary Figure 6**. **Gefitinib targets ZAP-70 in CLL cells**. (a) Primary ZAP-70- CLL cells were treated with DMSO or 10, 20, or 30 μM gefitinib for 1 hour then lysed. (b) Primary ZAP-70+ and ZAP-70- CLL cells were treated with DMSO or 10 μM gefitinib and lysed at 3, 6, 16, or 24 hours post treatment. (c) Primary ZAP-70+ CLL cells treated with DMSO or 1 μM gefitinib for 24 hours then lysed.(d) Densitometry of Syk and ZAP-70 immunoprecipitations in n=3 ZAP-70+ CLL samples after 1 hour of 30 μM gefitinib treatment and BCR stimulation. (e) Immunoprecipitation of Syk and ZAP-70 in a ZAP-70+ CLL sample pretreated with DMSO or 30 μM gefitinib, BCR stimulated, and lysed after 1 hour. Probed with anti-phospho-tyrosine antibody.


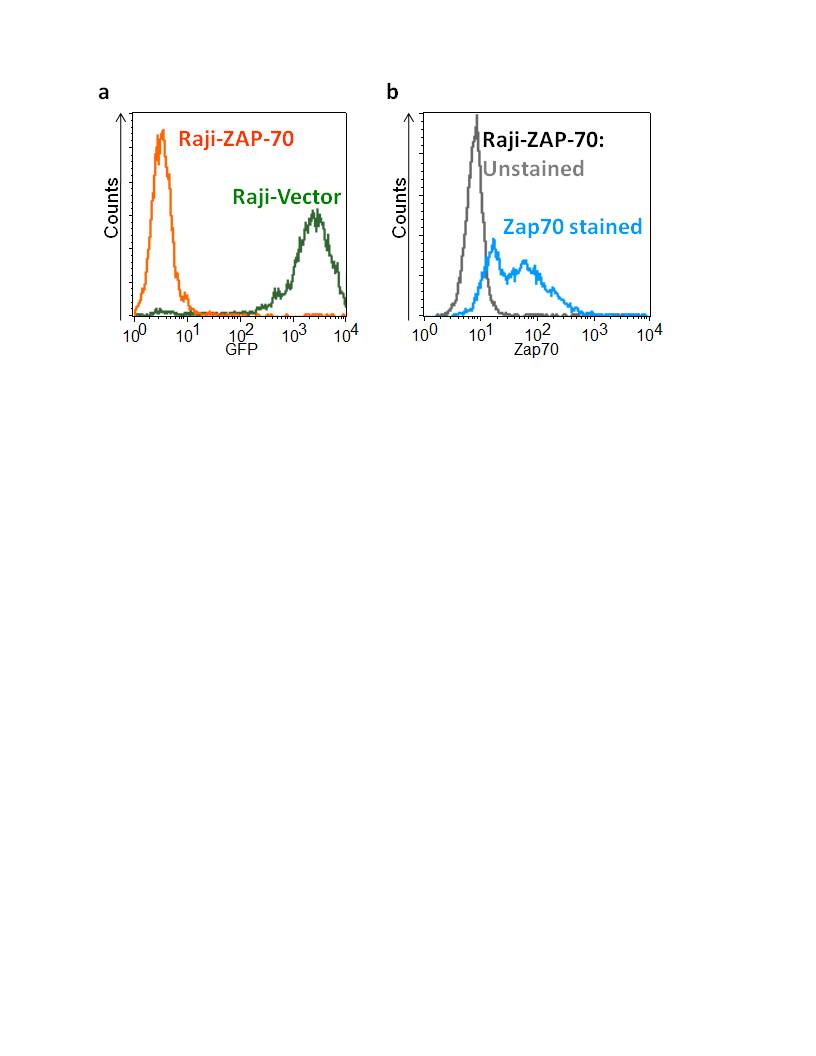


**Supplementary Figure 7. Raji-Vector cells express GFP and Raji-ZAP-70 cells express ZAP-70**. (a) Raji-Vector and Raji-ZAP-70 cells were analyzed by flow cytometry for the expression of GFP. (b) Raji-ZAP-70 cells were fixed, permeabilized, and stained with mouse IgG anti-ZAP-70-FITC, mouse IgG isotype control-FITC, or left unstained and analyzed by flow cytometry. Unstained and isotype control curves were identical.


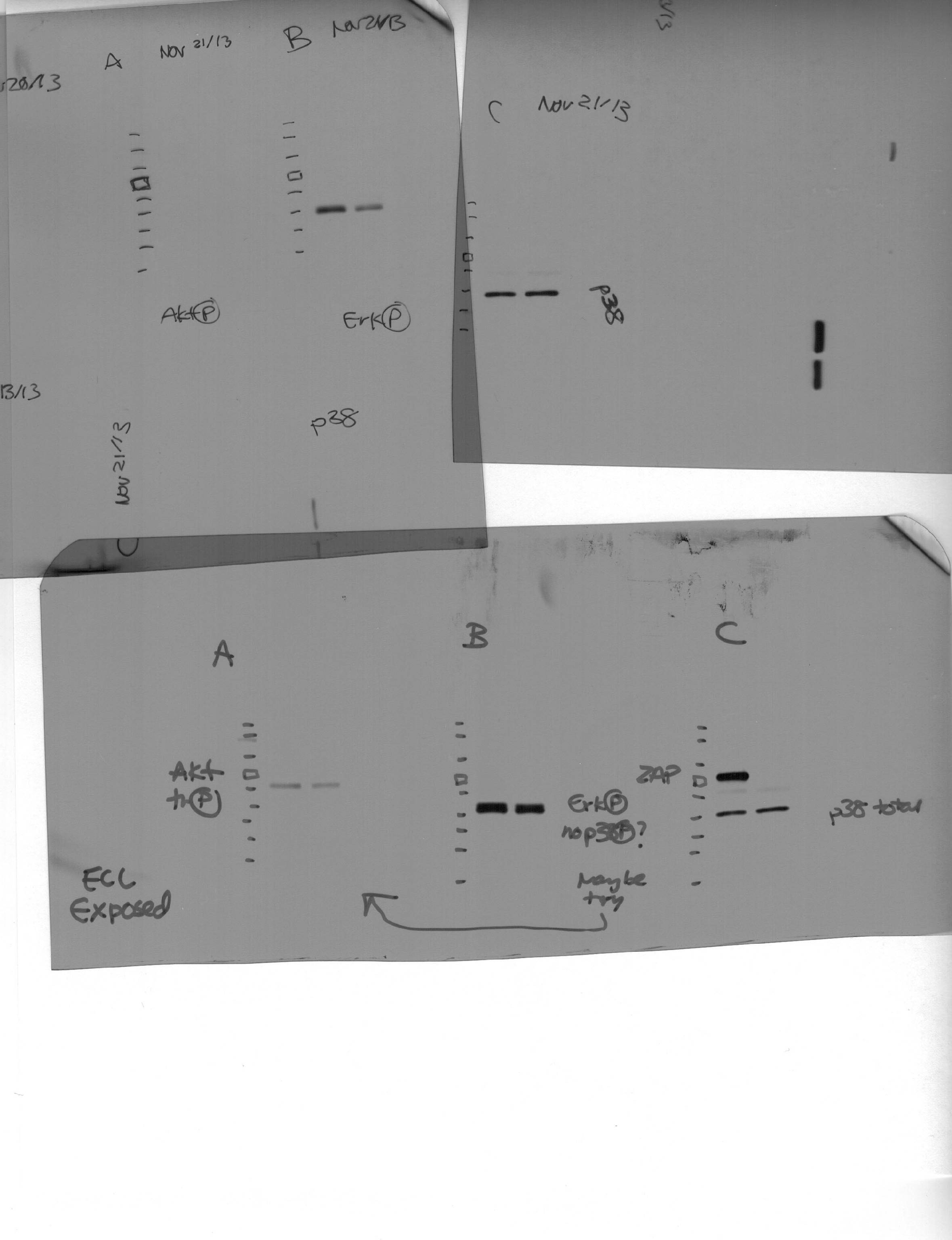

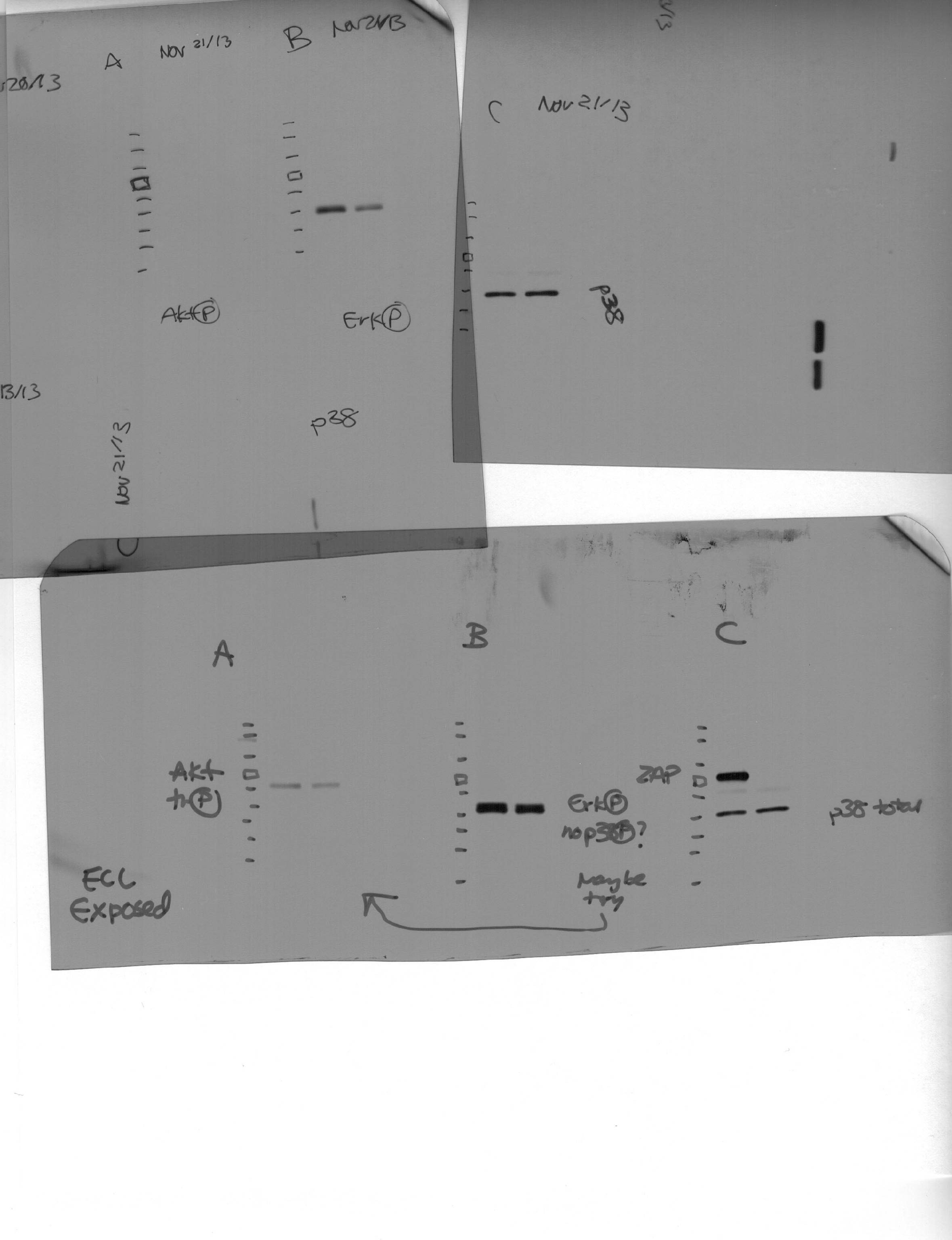

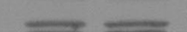

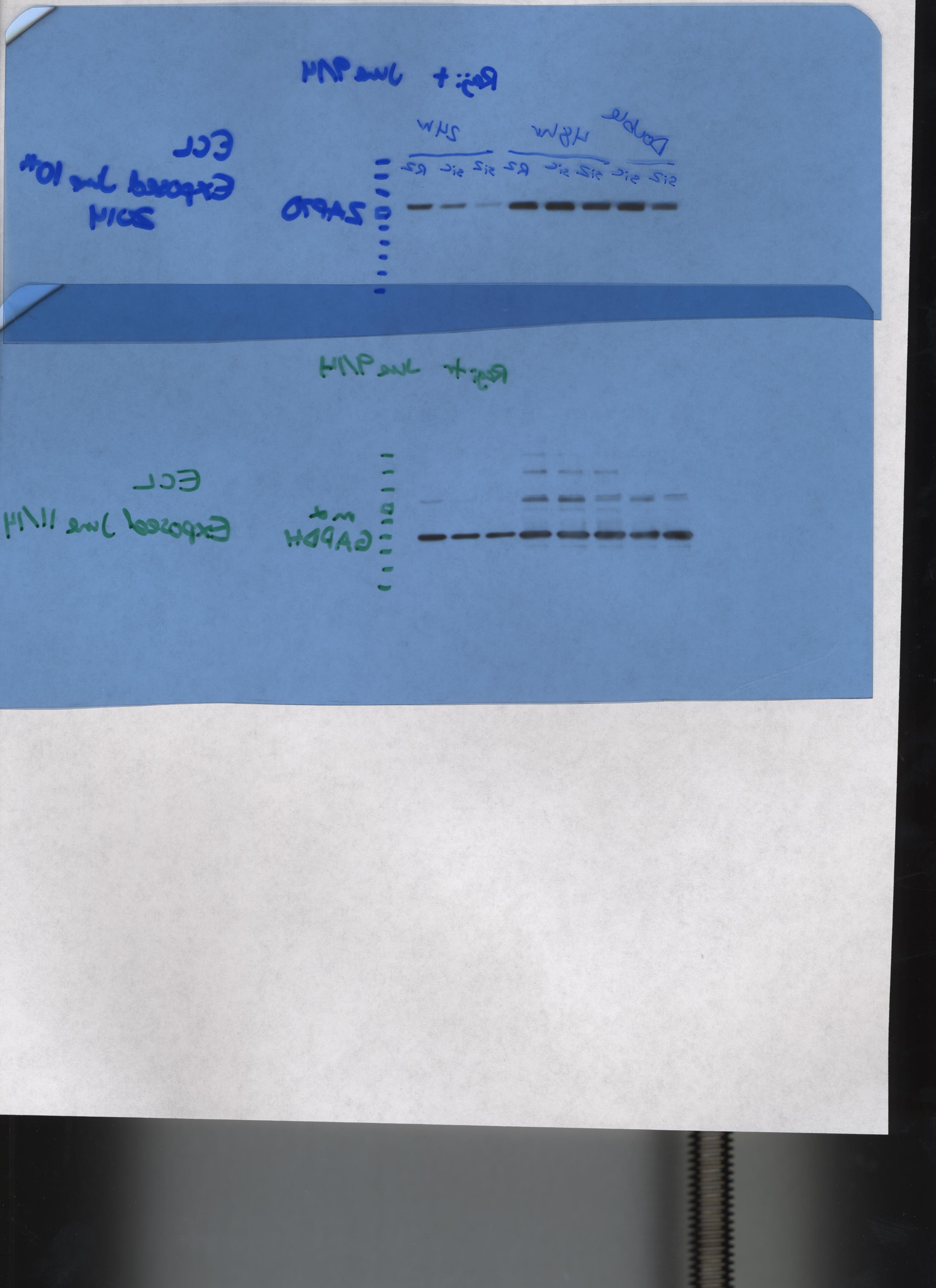


**a b**

Raji-Z Raji-V

ZAP-70

Erk-P

actin

siNTC siZAP-70


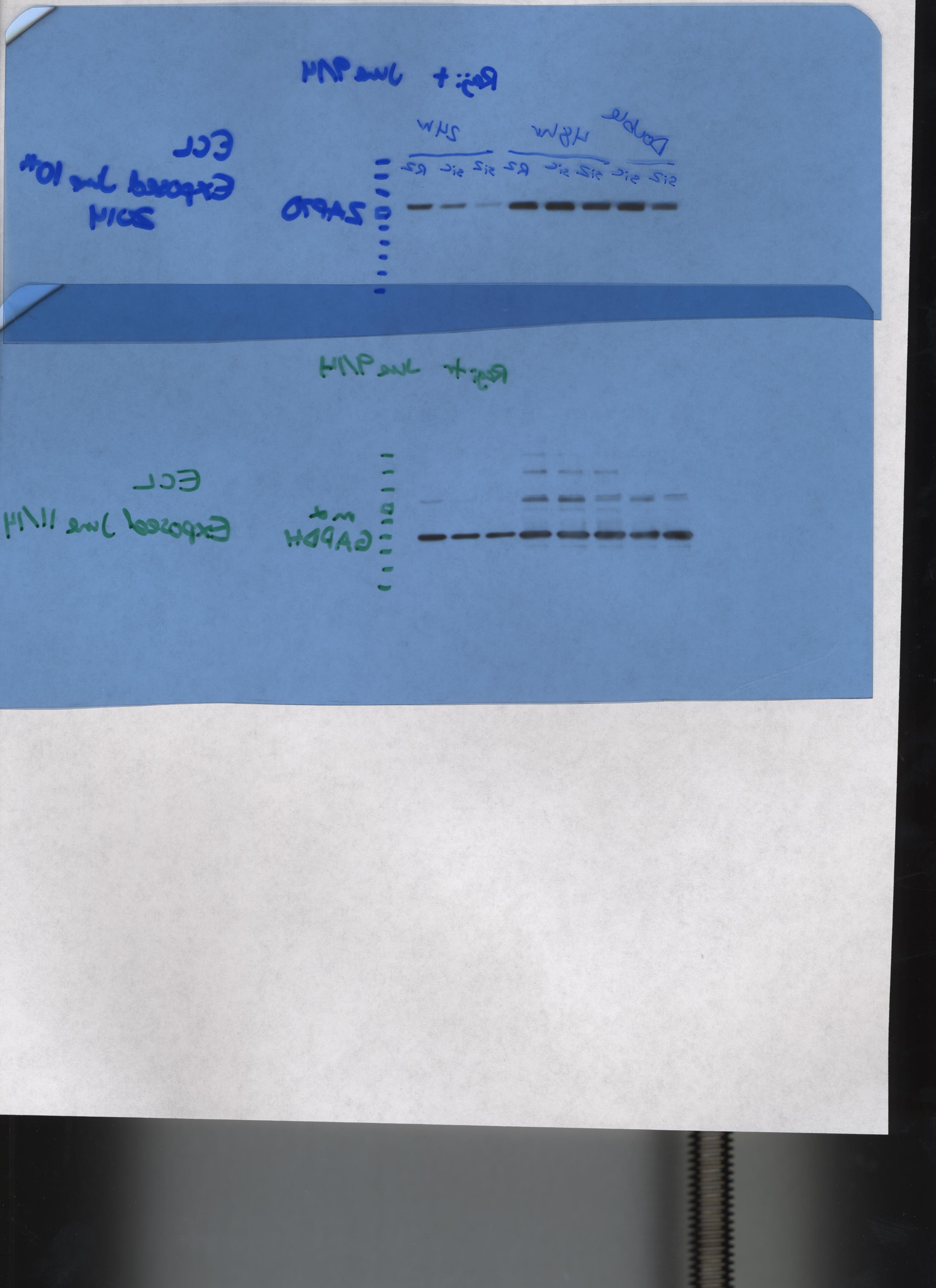


ZAP-70

Erk-P

Mcl-1

GAPDH


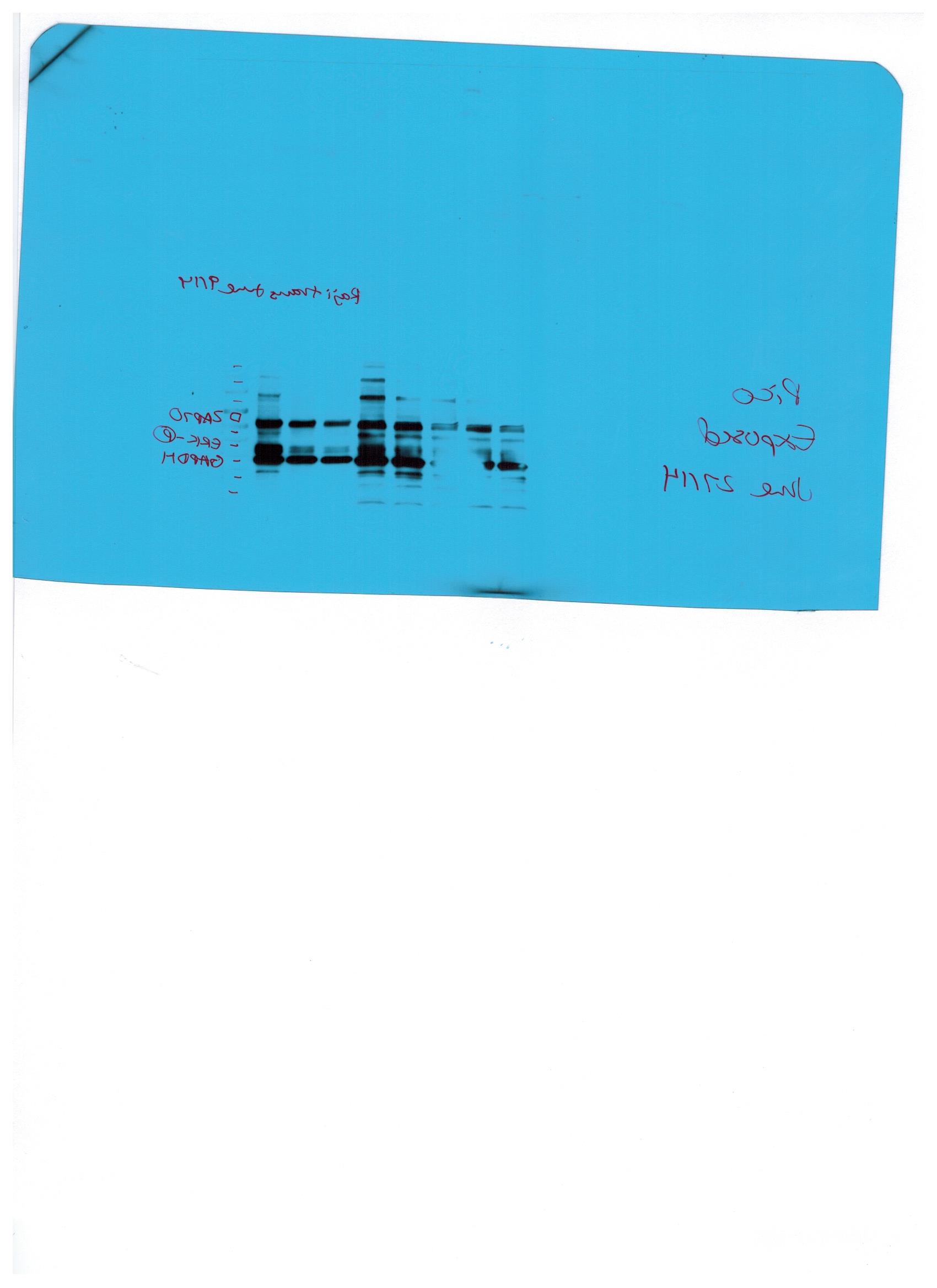


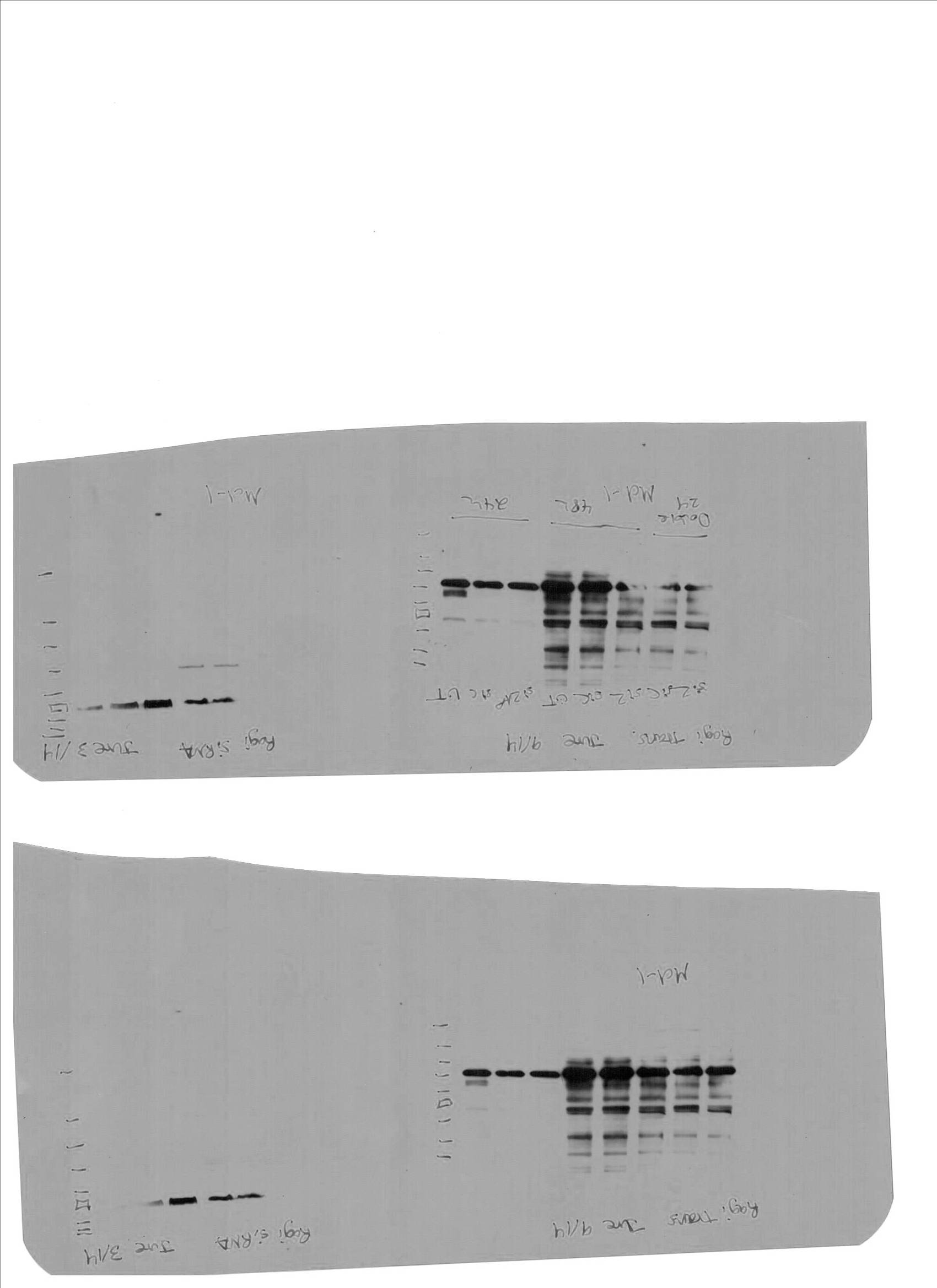


**Supplementary Figure 8. Knockdown of ZAP-70 decreases Erk activation and Mcl-1 expression**. Raji cells expressing vector alone (Raji-V) and Raji cells expressing ZAP-7- (Raji-Z) were lysed (a), or transfected with 30 pmol with siRNA against non-targeting control (siNTC) or ZAP-70 (siZAP70) and lysed 24 hours later (b). The lysate was western blotted for ZAP-70, phosphorylated Erk (Erk-P), and Mcl-1 and stripped and western blotted for actin and GAPDH as a loading controls.


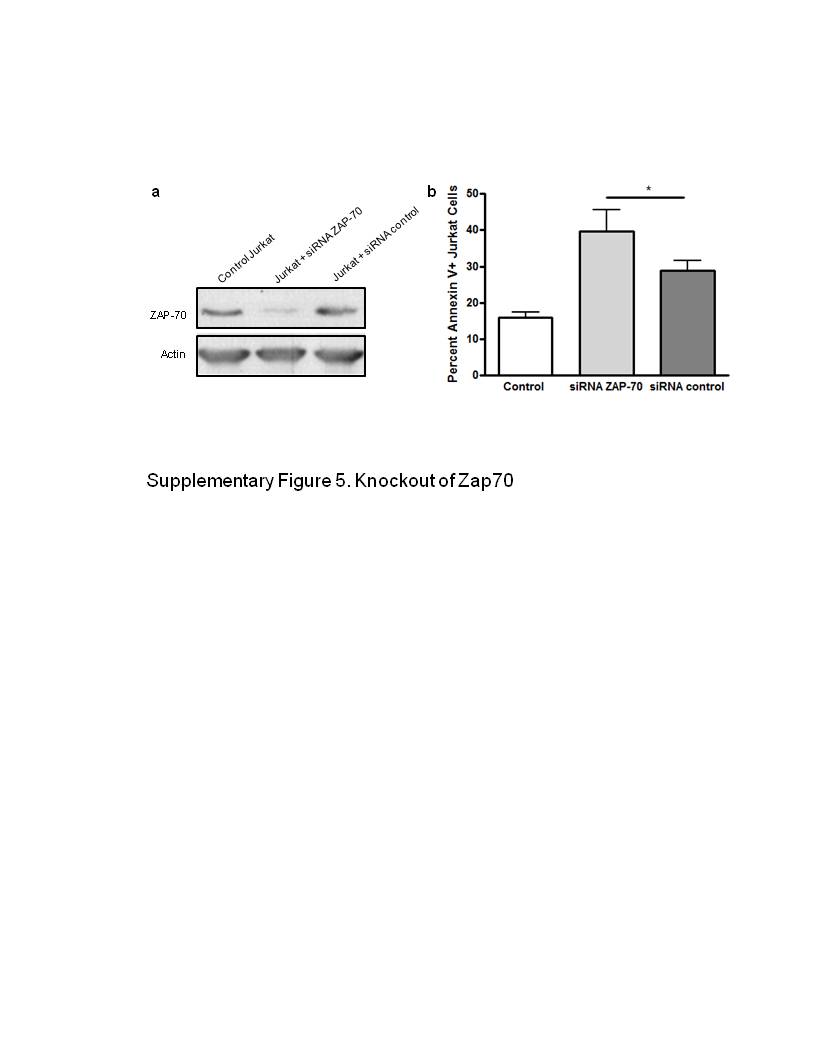


**Supplementary Figure 9. Knockdown of ZAP-70 is lethal in Jurkat cells**. Jurkat cells transfected with ZAP-70 siRNA, control siRNA, or left un-transfected were confirmed by western blot (a) and stained with Annexin V-FITC and analyzed for cell death by flow cytometry (b). Statistical test used was paired one-tailed t test.
